# The clinical value of adipokines in predicting the severity and outcome of acute pancreatitis

**DOI:** 10.1186/s12876-016-0514-4

**Published:** 2016-08-22

**Authors:** Andrius Karpavicius, Zilvinas Dambrauskas, Audrius Gradauskas, Arturas Samuilis, Kristina Zviniene, Juozas Kupcinskas, Gintautas Brimas, Artur Meckovski, Audrius Sileikis, Kestutis Strupas

**Affiliations:** 1 Center of Abdominal Surgery, Faculty of Medicine, Vilnius University, Santariskiu 2, LT-08661 Vilnius, Lithuania; 2 Department of Abdominal Surgery, Clinic of Surgery, Vilnius City Clinical Hospital, Antakalnio 57, LT-10207 Vilnius, Lithuania; 3 Institute for Digestive System Research, Lithuanian University of Health Sciences, Eiveniu 2, LT-50009 Kaunas, Lithuania; 4 Department of Surgery, Lithuanian University of Health Sciences, Eiveniu 2, LT-50009 Kaunas, Lithuania; 5 Department of Nursing and Fundamentals of Internal Medicine, Faculty of Medicine, Vilnius University, Antakalnio 57, LT-10207 Vilnius, Lithuania; 6 Radiology and Nuclear medicine Center, Faculty of Medicine, Vilnius University, Santariskiu 2, LT-08661 Vilnius, Lithuania; 7 Department of Radiology, Lithuanian University of Health Sciences, Eiveniu 2, LT-50009 Kaunas, Lithuania; 8 Department of Gastroenterology, Lithuanian University of Health Sciences, Eiveniu 2, LT-50009 Kaunas, Lithuania; 9 Center of General Surgery, Faculty of Medicine, Vilnius University, Siltnamiu 29, LT-04130 Vilnius, Lithuania

**Keywords:** Acute pancreatitis, Severity, Prognosis, Prediction, Adipokines, Resistin, Visfatin, Leptin, Adiponectin

## Abstract

**Background:**

Recent data shows that patients with severe acute pancreatic might benefit from early intensive therapy, enteral nutrition and timely transfer to specialized centers. The early prophylactic use of antibiotics in AP remains controversial. The role and need for new markers in stratification of acute pancreatitis is also uncertain. This study aims to evaluate the prognostic usefulness of adipokines in prediction of the severity and outcome of acute pancreatitis (AP).

**Methods:**

Prospective study was conducted in four clinical centers. The diagnosis and severity assessment of AP was established according to the revised 2012 Atlanta classification. Adipokines, IL-6 and CRP levels were measured at admission and on 3rd day of hospital stay and compared with the control group. The predictive accuracy of each marker was measured by area under the receiver operating curve.

**Results:**

Forty healthy controls and 102 patients were enrolled in to the study. Twenty seven (26.5 %) patients had mild, 55 (53.9 %) - moderate and 20 (19.6 %) - severe AP. Only resistin (cut-off value 13.7 ng/ml) and IL-6 (cut-off value 473.4 pg/ml) were reliable early markers of SAP. IL-6 with cut-off value of 157.0 pg/ml was a predictor of necrosis. The peripancreatic necrosis volume of 112.5 ml was a marker of SAP and 433.0 ml cut-off value could be used to predict the need of interventions.

**Conclusions:**

The prognostic value of adipokines in AP is limited. Only admission resistin levels could serve as an early predictor for SAP.

The Lithuanian Regional Ethics Committee approved the study protocol (permission No. L-12-02/1/2/3/4) and all the patients and the control group provided written informed consent.

## Background

Acute pancreatitis (AP) is a disease with highly variable clinical course. Majority of patients have mild and self-limiting disease, but the mortality rate of patients with severe and complicated forms can reach up to 30 % [1–4]. Non-complicated forms of AP can be treated in smaller regional hospitals, but the patients with the severe course of AP should be timely transferred to the high volume centers [5]. It has been reported that hospitals treating higher number of AP cases have better clinical outcomes [6, 7]. A number of predictive markers and scoring systems were introduced in to clinical practice for early prediction of severe AP (SAP), local or systemic complications and mortality. The importance of the early prognosis of the course of the disease was shown in some studies. Data revealed that very low mortality rates are associated with early recognition of SAP patients and adequate intensive therapy [8]. Enteral nutrition, if started at least within 48 h after admission, can reduce the incidence of complications and can contribute to an increased rate of survival [5]. The early prophylactic use of antibiotics in AP remains controversial. American College of Gastroenterology and IAP/APA guidelines both recommend against antibiotic use [9–11]. But according to the newest Japanese guidelines for the management of acute pancreatitis based on recent meta-analysis, the prophylactic administration of antibiotics in SAP and necrotizing pancreatitis may improve the prognosis, if carried out within 72 h after onset of AP [5, 12, 13]. So, the discussion about the early use of antibiotics is still open, because it may turn out that they are useful for selective patient group.

The adequate management of the AP patients within first 2 3 days could be of vital importance and determine the future prognosis. Based on the revised Atlanta 2012 classification moderately severe acute pancreatitis is defined by the presence of transient organ failure, local complications or exacerbation of co-morbid disease. Severe acute pancreatitis is defined by persistent organ failure, that is, organ failure >48 h. Thus in the real clinical setting it is very difficult to determine the severity of the disease within the first hours after admission, because it is not known whether the patient would have transient, persistent organ failure or no organ failure at all. It is suggested that initially the patient should be classified and treated as potentially having SAP, if he/she does not have mild pancreatitis on admission [14]. However, this is only possible in specialized, high volume centers, whereas smaller regional hospitals could face certain difficulties implementing this strategy.

Currently prognosis of the AP is largely based on clinical scores, such as APACHE II, BISAP and many others. The main problem is that all of them are multifactorial and rather uncomfortable for everyday use, so there is a great stimulus for seeking new accurate single prognostic marker. C-reactive protein (CRP) is the most widely explored and described single predictor for disease severity and pancreatic necrosis. However, its concentration reaches a peak on 3rd day of the disease, so it has a greatest prognostic value approximately 48 h after the onset of the symptoms. IL-6 is also introduced in clinical practice and is approved as a reliable prognostic marker in many countries.

The predictive value of adipokines, such as leptin, adiponectin, resistin and visfatin, is less explored. Adipokines are cytokines produced in white adipose tissue as well as in peripancreatic fat and involved in inflammatory response. Increase of fatty tissue due to obesity is associated with the amplified systemic inflammatory response in AP; furthermore it can be used as a prognostic factor for mortality, local, systemic complications and severity of AP [15]. Peripancreatic fat necrosis in acute pancreatitis is associated with the development of SAP, multiple organ failure and mortality [16, 17]. It is hypothesized that peripancreatic necrosis can cause the massive release of adipokines into the bloodstream, so adipokines can serve as predictors of clinical course and complications of acute pancreatitis.

The significant differences of resistin, visfatin, leptin and adiponectin concentrations between mild AP (MAP) and SAP patients were found in some older studies, but all of them were very different in their methodology, diagnostic criteria, classification and evaluation of AP [18–24]. Furthermore, in 2012 the Atlanta classification of AP was revisited and a new form of moderately severe AP was identified [14]. Therefore the criteria of SAP became more stringent and the cut off values for adipokines, as well as for CRP and IL-6 must be recalculated.

## Methods

### Study design and patient population

Our study was conducted in four Lithuanian hospitals during the period between April 2012 and March 2015. The Regional Ethics Committee approved the study protocol (permission No. L-12-02/1/2/3/4) and all the patients and the control group provided written informed consent.

The diagnosis of AP was established according to the revised Atlanta 2012 classification and based on the presence of at least two of the three following features: abdominal pain characteristic of acute pancreatitis, serum amylase level ≥3 times up the upper limit of normal and characteristic findings of AP on abdominal computerized tomography scan.

All patients admitted to the hospitals with a diagnosis of acute pancreatitis and onsets of the symptoms within last 72 h were included in this study. Pregnant women, patients with the history of necrotizing pancreatitis and underlying chronic pancreatitis were excluded from this study.

Each patient’s age, sex, etiological factor, body mass index (BMI), presence of organ failure and local complications, interventions, in-hospital mortality and length of hospital stay were recorded.

According to the revisited Atlanta classification, based on organ failure, all the AP patients retrospectively were classified as mild, moderate or severe AP cases.

Percutaneus drainage, endoscopic, laparoscopic and open necrosectomy were classified as interventions. No interventions were performed in the conservative management group. Indications for interventions were clinical suspicion of or radiologically proven infected pancreatic or peripancreatic necrosis with clinical deterioration or ongoing organ failure several weeks after the onset of the AP, preferably when necrosis was walled-off [11].

The prognostic value of adipokines in the prediction of the development of pancreatic necrosis, severe course of the disease, need for surgical intervention and mortality were the primary outcome measures in this study. The development of peripancreatic necrosis was the secondary measure.

### Blood samples

Peripheral blood samples from AP patients were obtained at the day of the admission and after 48–72 h (3rd day). The blood samples of the control group were obtained only once. All the samples were centrifuged and stored at −20 °C until analysis. Blood sample analysis was performed at the Center of Laboratory Medicine, Vilnius University. Adipokines and IL-6 serum concentrations were measured using ELISA kits (DIAsourceImmunoAssays SA/Adiponectin, IBL/International Leptin Elisa, DIAsourceImmunoAssays SA/Resistin ELISA, BioVendor Human Visfatin (Nampt) ELISA and DIAsourceImmunoAssays SA/IL6) according to the manufacturer’s instructions. Compact microplate processor Gemini (Stratec Biomedical AG) was used. Plasma levels of CRP and other tests were measured in accordance with hospitals laboratory routine.

### CT scan

Contrast enhanced CT (CECT) scans were performed for patients with acute pancreatitis no earlier than the 3rd day and no later than the 7th day after the onset of symptoms. All CECT examinations were performed in four centers:

1. Vilnius University Hospital “Santariskių Klinikos”
2. Vilnius City Clinical Hospital
3. Republican Vilnius University Hospital
4. Hospital of the Lithuanian University of Health Sciences “Kauno Klinikos”

All examinations were performed on multidetector CT scanners (GE VCT, GE Light Speed Pro and Toshiba Aquilion) and covered abdominal region and pelvic region, if required. Standard pancreatic scanning protocol was used with late arterial and portovenous phases. CT scans were retrospectively and independently reviewed on workstations (GE Advanced WorsktationVolumeShare 5 (AW4.6)) by two experienced abdominal radiologists who were unaware of presenting signs and symptoms or of patient outcomes. Each case was independently assessed by both observers using the modified CT severity index (MCTSI). Possible pancreatic necrosis and their extent, peripancreatic fluid collections and extrapancreatic findings (pleural effusion, ascites, parenchymal, vascular and GI tract complications) were evaluated. In the cases of disagreement of indexes between two radiologists consensus was reached after secondary review of CT scans by the same radiologists and discussion. Up to three biggest peripancreatic fluid collections were measured in three perpendicular dimensions (in cm). The simplified formula of an ellipsoid was used (length x width x thickness/2) to calculate the volume of the peripancreatic fluid collections. This formula enables quick and easy calculation of the volume and is widely used in radiology [25–27].

### Statistical analysis

Statistical analysis was performed using R v. 3.2.0 package. Categorical variables were expressed as absolute numbers and percent. For the association between two variables Pearson's chi-square test or Fisher's exact test were applied, as appropriate. Continuous variables were expressed as mean ± standard deviation (SD) and median ± interquartile ranges (IQR). Normality of the variables was checked by Shapiro-Wilk statistic. All variables, except age, were not distributed according to the normal distribution; therefore nonparametric hypotheses were tested to detect significant differences between selected categories. ROC curves, area under the curve (AUC) and optimal cut off values were calculated using R plugin pROC [28]. AUC was calculated using the 95 % confidence interval (CI). A *p* value less than 0.05 was considered statistically significant.

## Results

### Patients’ characteristics

**Fig. 1:**
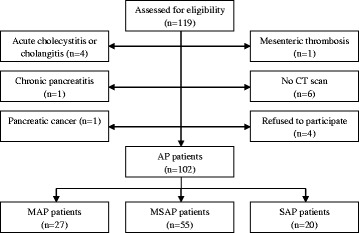
The flowdiagram for patient’s selection. Abbreviations: CT – Computed Tomography; AP – Acute Pancreatitis; MAP – Mild Acute Pancreatitis; MSAP - Moderately severe acute pancreatitis; SAP – Severe Acute Pancreatitis

During the study period 119 of AP patients were prospectively assessed for possible inclusion in the study. Seventeen patients were excluded for various reasons (Fig. 1). In the final analysis 102 of AP patients (50 males and 52 females, mean age 55.7 ± 18.1 years) were included. Mean time after onset of the symptoms was 20.3 ± 13.8 h. The main etiological factors of AP were biliary stones (42.2 %) and alcohol (35.3 %). Necrosis of the pancreas during CECT was detected in 60 (58.8 %) patients and the peripancreatic necrosis was present in 67 (65.7 %) cases. Ninety two (90.2 %) patients were treated conservatively and 10 (9.8 %) underwent the interventions. Only percutaneus drainage was used in one case (10.0 %), four patients (40.0 %) underwent only open necrosectomy and the last five operated patients got combined interventions (50.0 %); Mean length of hospital stay was 20.8 ± 28.3 days. Five patients died during hospitalization (mortality rate – 4.9 %).

According to the revisited Atlanta classification, based on organ failure, 27 (26.5 %) of all patients had mild, 55 (53.9 %) - moderately severe, and 20 (19.6 %) - severe AP (Fig. 1). The main differences in the clinical and biochemical characteristics between SAP and milder forms of the AP are shown in Table 1.

**Table 1:** Differences in clinical course of mild/moderately severe and severe AP

	Mild and moderately severe AP	Severe AP	*P* value*
	(*n* = 82)	(*n* = 20)	
Age	55.90 ± 19.02	55.00 ± 14.20	0.814
Sex			
Male (%)	38 (46.3)	12 (60.0)	
Female (%)	44 (53.7)	8 (40.0)	
BMI	27.99 ± 7.54	31.07 ± 10.02	**0.041**
Adiponectin μg/ml (1st day)	11.10 ± 9.58	7.91 ± 10.07	0.446
Adiponectin μg/ml (3rd day)	10.04 ± 9.14	8.64 ± 6.44	0.870
Leptin ng/ml (1st day)	7.21 ± 11.83	4.17 ± 8.14	0.397
Leptin ng/ml (3rd day)	2.33 ± 3.85	0.84 ± 6.03	0.533
Visfatin ng/ml (1st day)	4.15 ± 5.45	5.42 ± 4.74	0.179
Visfatin ng/ml (3rd day)	2.94 ± 4.58	7.34 ± 5.68	0.059
IL-6 pg/ml (1st day)	133.00 ± 350.47	635.95 ± 634.45	**0.000**
IL-6 pg/ml (3rd day)	94.97 ± 323.86	545.31 ± 574.17	**0.000**
Resistin ng/ml (1st day)	10.70 ± 8.65	20.20 ± 31.75	**0.000**
Resistin ng/ml (3rd day)	11.67 ± 14.87	40.75 ± 28.27	**0.000**
CRP mg/ml (1st day)	9.54 ± 64.79	16.15 ± 74.86	0.220
CRP mg/ml (3rd day)	180.30 ± 224.87	377.13 ± 91.39	**0.000**
SOFA (1st day) (score)	1 ± 2	3 ± 3	**0.000**
SOFA (3rd day) (score)	1 ± 2	4 ± 2	**0.000**
MCTSI (score)	6 ± 4	8 ± 2	**0.000**
Pancreatic necrosis (%)	40 (48.8)	20 (100)	**0.000**
Peripancreatic necrosis volume (ml) (n)	31.50 ± 518.50 (48)	731.00 ± 2141.50 (19)	**0.000**
Need for surgery (%)	2 (2.4)	8 (40.0)	**0.000**
Hospital stay (d)	11.00 ± 7.00	30 ± 22.75	**0.000**
Number of deaths (%)	0 (0.0)	5 (25.0)	**0.000**

*Abbreviations*: *AP* Acute Pancreatis, *BMI* Body Mass Index, *IL-6* Interleukin-6, *CRP* C-Reactive Protein, *SOFA* The Sequential Organ Failure Assessment score, *MCTSI* Modified Computed Tomography Severity Index

Age was expressed as mean ± standard deviation (SD); sex, pancreatic necrosis, need for surgery and number of deaths by percents, other variables - as median ± interquartile ranges (IQR)

*Significant in bold

Forty healthy persons were included as a control group: 17 males and 23 females with the mean age of 54.3 ± 16.1 years and BMI of 27.9 ± 4.7 kg/m^2^. No significant differences were noted between the AP patients and control group in terms of their gender, age or BMI.

#### Serum resistin, visfatin and IL-6 values are significantly higher in AP patients than in controls

Median admission serum adiponectin levels were higher in AP group (median 10.7 μg/ml, Q1-Q3 6.8–16.8 μg/ml) than in controls (median 8.3 μg/ml, Q1-Q3 5.6–12.3 μg/ml), *p* > 0.05. Median admission serum leptin levels were higher in AP group (median 6.7 ng/ml, Q1-Q3 2.8–14.5 ng/ml) than in controls (median 4.0 ng/ml, Q1-Q3 1.5–8.5 ng/ml), *p* > 0.05. Median admission serum resistin levels were higher in AP group (median 12.6 ng/ml, Q1-Q3 7.4–18.2 ng/ml) than in controls (median 5.4 ng/ml, Q1-Q3 4.5–6.7 ng/ml), p < 0.05. Median admission serum visfatin levels were higher in AP group (median 4.7 ng/ml, Q1-Q3 2.1–7.4 ng/ml) than in controls (median 1.6 ng/ml, Q1-Q3 1.2–2.2 μg/ml), *p* < 0.05. Median admission serum IL-6 levels were higher in AP group (median 194,3 pg/ml, Q1-Q3 39,8–508,8 pg/ml) than in controls (median 1.5 pg/ml, Q1-Q3 0.3–7.0 pg/ml), *p* < 0.05.

#### Resistin and IL-6 are useful early markers for prediction of severity in AP

**Fig. 2:**
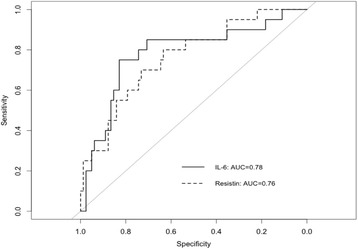
Prognostic value of admission resistin and IL-6 levels for development of SAP. IL-6 cut-off value 473.4 pg/ml predicts SAP (sens. 82.9 %, spec. 75.0 %, PPV 51.7 %, NPV 93.2 %, AUC 0.78). Resistin cut-off value 13.7 ng/ml predicts SAP (sens. 63.4 %, spec. 80.0 %, PPV 34,8 %, NPV 92,9 %, AUC 0.76). Abbreviations: IL-6 – Interleukin-6; AUC - area under the curve

Median admission and 3rd day resistin, IL-6 and 3rd day CRP values were significantly higher in SAP group when compared with other patients. No significant differences were noted for admission and 3rd day adiponectin, leptin, visfatin and admission CRP values between SAP and other patients (Table 1). The ROC analysis applied for early SAP prediction showed significant results only for admission resistin and IL-6 (Fig. 2). The detail results are presented in Table 2.

**Table 2:** Admissions levels of resistin and IL-6 as predictors of SAP

	AUC	95 % CI	Cut-off	Sens., %	Spec., %	PPV	NPV
IL-6 Admission	0.78	0.6596–0.9075	473.4	82.9	75.0	51.7	93.2
IL-6 3rd day	0.82	0.7322–0.9117	119.9	54.9	100.0	35.1	100.0
Resistin Admission	0.76	0.6462–0.8782	13.7	63.4	80.0	34.8	92.9
Resistin 3rd day	0.89	0.8232–0.9597	23.9	79.3	85.0	50.0	95.6
CRP Admission	0.59	0.4542–0.7238	4.4	35.4	90.0	25.4	93.5
CRP 3rd day	0.79	0.6932–0.8873	301.1	70.7	80.0	40.0	93.5

*Abbreviations*: *IL-6* Interleukin-6, *AUC* area under the curve, *CI* confidence interval, *Sens* sensitivity, *Spec* specificity, *PPV* positive predictive value, *NPV* negative predictive value

#### Admission adipokine levels can’t predict development of necrosis, need for interventions and mortality

The ROC analysis applied to predict the development of necrosis on admission showed no significant results for adipokines. Only admission IL-6 with a cut-off 157.0 pg/ml could be used for early prediction of necrosis (sensitivity 75.0 %, specificity 67.1 %, AUC 0.72). The ROC analysis applied to predict the need for interventions and mortality on admission also showed no statistically significant results.

#### The peripancreatic necrosis is associated with the development of SAP

Median volume of the peripancreatic necrosis was lower in mild and moderately severe AP (median 31.5 ml, Q1-Q3 0–518.5 ml) than in SAP (median 731.0 ml, Q1-Q3 432.5–2574.0 ml), *p* < 0.05. The analysis of the ROC curves demonstrated, that the cut-off value of 112.5 ml is associated with SAP (sensitivity 61.0 %, specificity 95.0 %, AUC 0.80) and 433.0 ml cut-off value is associated with the need of intervention (sensitivity 68.5 %, specificity 100 %, AUC 0.87).

## Discussion

According to the revised Atlanta 2012 classification diagnosis of SAP is based on organ failure and can be determined only after 48 h from admission [14]. These 2 days can be fatal for the AP patient, so early prediction of SAP remains very important. Optimal marker should help to separate the milder forms of the disease from severe ones even on admission. So, all previously published results about prognostic possibilities of the different markers after 48 h from admission are not clinically important and we need to focus only on the 1st day data. Only the early predictor could help to make the decision about the patient’s needs to be transferred to the high volume center timely, enteral nutrition and maybe prophylactic administration of antibiotics use in early phase of AP.

Prognostic value of adipokines in AP patients has been noticed some years ago. Experimental studies with rats started in 2002 and demonstrated significant differences of leptin concentrations between AP patients and controls, and between acute edematous and acute necrotizing pancreatitis groups [29–31]. Although in some other clinical studies the prognostic value of leptin on pancreatic necrosis was proven [23], according to our data leptin is not suitable for predicting SAP, development of necrosis, need for interventions and mortality. However, this data replicates the results of some previously published studies [24, 32, 33].

Only one study published in 2009 has shown adiponectin to be a valuable prognostic marker of the SAP, whereas, serum adiponectin levels from 1st to 3rd day were significantly lower for patients with SAP than for those with MAP [19]. According to our data and other studies, adiponectin is also not a good marker for the prediction of AP course [33]. There was no significant difference of adiponectin concentrations even between the AP patients and the healthy controls in our study.

Some promising results related to the resistin and visfatin prognostic value were published in 2007–2011 by Shaffler’s group [20–22]. This study has shown that both adipokines concentrations had significant differences between MAP and SAP groups, they correlate with severity of disease, need for interventions and outcome. Resistin and visfatin are also good markers for peripancreatic necrosis. The cut-off values of 11.9 ng/ml and 1.8 ng/ml respectively allows the higher ranges of radiological scores prediction. These results are consistent with another study, published in 2010, which demonstrates, that both adipokines may be possibly used for AP prognosis and disease monitoring [34].

Despite the above mentioned data, our results on visfatin did not meet the expectations. The admission levels of visfatin hasn’t shown any significant value for predicting the development of SAP or necrosis, need for interventions and mortality, although the significant differences of visfatin concentrations between AP and healthy controls were observed during the study.

Interestingly, we observed the statistically significant differences of admission resistin concentrations between all compared groups. The cut-off value of 13.7 ng/ml predicts SAP (sensitivity 63.4 %, specificity 80.0 %, AUC 0.76) on admission. This is consistent with the results presented by *Schaffler* group which demonstrated that admission resistin cut-off value of >11.9 ng/mL can serve as a positive predictor of a Balthazar score >3 and Necrosis score >2 [21]. Compared to the last published meta-analysis and other publications on BISAP (cut-off ≥3, sensitivity 51–62 %, specificity 72–91 %, AUC 0.74–0.87) and APACHE II (cut-off ≥8, sensitivity 81–83 %, specificity 59–66 %, AUC 0.78–0.82) scores, the result of resistin as a single predictor for SAP is acceptable [35, 36]. Nevertheless, it is not as universal as IL-6, which according to our data can predict SAP on admission with the cut-off 473,4 pg/ml (sensitivity 82,9 %, specificity 75,0 %, AUC 0,78) as well as necrosis (cut-off 157.0 pg/ml, sensitivity 75.0 %, specificity 67.1 %, AUC 0.72). Our cut-off value of IL-6 as the predictor of SAP is similar to the results of *Nieminem* group published in 2014 (cut-off 501.6 pg/ml, sensitivity 48.0 %, specificity 93.5 %, AUC 0.81) [37]. Though, much lower cut-off values of IL-6 for prediction of necrosis are demonstrated by the other authors – in the range of 50–83.3 pg/ml (with sensitivity 84–94 %, specificity 72–73 %, AUC 0.79–0.86) [38, 39]. The discrepancy between earlier published studies and our own results could be explained by the fact that the other researchers assessed only the necrosis of pancreas and didn’t assess the peripancreatic necrosis as the possible source of inflammatory cytokines. The data from other authors and our results show the importance of the peripancreatic necrosis in pathogenesis of SAP [17].

Our study has shown that the value of adipokines in predicting the course and outcome of AP was rated too good. Only resistin can be used for early AP course prediction. The value of peripancreatic necrosis, as the main possible source of resitin in the course of AP haven’t been studied enough. According to our data even 112.5 ml volume of peripancreatic necrosis is associated with SAP (sensitivity 61.0 %, specificity 95.0 %, AUC 0.80). Similar results comes from *Meyrignac* group study [17]. Although peripancreatic necrosis is assessed only on CT scan and it’s prognosis on AP course is too late, surgeons should be interested in a fact, that 433.0 ml cut-off value of the peripancreatic necrosis is associated with a greater need for intervention (sensitivity 68.5 %, specificity 100 %, AUC 0,87). So there is a great stimulus to initiate a new study trying to find the relationship between resistin, peripancreatic necrosis, acute pancreatitis course, complications and outcomes.

Despite the large amount of prognostic AP studies, optimal early marker has not been found yet. Probably no physician would send the AP patient to intensive care unit or other specialized center without other clinical findings based only on the high concentrations of the prognostic markers. Errors in assessing the AP patient’s condition in early phase could be dramatic. That is why the evaluation of the AP patient’s status must be complex and based on revised Atlanta classification recommendations, prognostic markers, scores and, of course, on each physician’s clinical experience.

## Study limitations

Like many other studies focusing on the research of the prognostic biochemical markers our study also has some methodological and technical limitations. Although the study was multicenter, the number of patients in SAP group was significantly lower than in MAP + MSAP group. From 102 included AP patients only 20 had the severe course of the disease. Therefore, the relatively small number of patients with SAP was compared to the group of 82 patients with MAP and MSAP.

Because of the conservative treatment strategy of AP in last decade the number of interventions and deaths decreased significantly. In our cohort, 10 out of 102 patients required interventional treatment and five patients died during the hospitalization. The lack of the patients was the reason that we couldn’t separate AP by the type of the necrosis. Only three patients had pancreatic necrosis without signs of peripancreatic necrosis. Another study limitation is difficulties in distinction by CECT between acute peripancreatic fluid collection and acute necrotic collection in the 1st week of AP. A diagnosis of peripancreatic necrosis based CECT findings often cannot be made specifically but can be suspected when slightly heterogeneous peripancreatic collections are seen. After 1 week from onset, the collection usually becomes clearly heterogeneous, and peripancreatic necrosis can be diagnosed on CECT images.

As several previously published studies report, adipokines have a good predictive value for the AP course of population with obesity. In this study we also tried to divide AP patients according to their BMI, but the groups were not homogenic and the number of patients in some groups was not satisfactory for analysis. So, for now we could not assess adipokines in predicting severity and outcome of AP among people suffering from obesity. Of course, this kind of investigation could be useful in routine clinical practice for the prediction of AP course and outcome among high risk (high BMI) patients. That is why we’ll try to accomplish this idea in the future with sufficient number of patients.

## Conclusions

Resistin and IL-6 cut-off values 13.7 ng/ml and 473.4 pg/ml could be used as an early markers of severe AP as well as the peripancreatic necrosis volume of 112.5 ml. The IL-6 cut-off value 157.0 pg/ml predicts necrosis and 433.0 ml cut-off value of peripancreatic necrosis is associated with a greater need for intervention.

There is no optimal early marker for AP severity stratification yet. The evaluation of the AP patient’s status at first few hours after admission must be complex and only then the right decision could be made.

## Abbreviations

AP: Acute pancreatitis
APACHE II: Acute physiology and chronic health evaluation II
AUC: Area under the curve
BISAP: Bedside index of severity in acute pancreatitis
BMI: Body mass index
CECT: Contrast enhanced computed tomography
CI: Confidence interval
CRP: C-reactive protein
CT: Computed tomography
IL-6: Interleukin 6
IQR: Interquartile ranges
MAP: Macute pancreatitis
MCTSI: Modified computed tomography severity
MSAP: Moderately severity acute pancreatitis
NPV: Negative predictive value
PPV: Positive predictive value
ROC: Receiver operating characteristic
SAP: Severe acute pancreatitis
SD: Standard deviation
Sens.: Sensitivity
SOFA: The sequential organ failure assessment score
Spec.: Specificity
